# Body perception disturbances in women with pregnancy-related lumbopelvic pain and their role in the persistence of pain postpartum

**DOI:** 10.1186/s12884-021-03704-w

**Published:** 2021-03-18

**Authors:** Nina Goossens, Inge Geraerts, Lizelotte Vandenplas, Zahra Van Veldhoven, Anne Asnong, Lotte Janssens

**Affiliations:** 1 REVAL Rehabilitation Research Center, UHasselt - Hasselt University, Agoralaan A, 3590 Diepenbeek, Belgium; 2 Department of Rehabilitation Sciences, KU Leuven, ON IV Herestraat 49 - box 1510, 3000 Leuven, Belgium; 3 Department of Gynaecology and Obstetrics, University Hospitals Leuven, Herestraat 49, 3000 Leuven, Belgium

**Keywords:** Lumbopelvic pain, Body perception, Disability, Pain intensity, Pregnancy, Postpartum, Primipara

## Abstract

**Background:**

Lumbopelvic pain (LPP) is common during pregnancy and can have long-lasting negative consequences in terms of disability and reduced quality of life. Therefore, it is crucial to identify women at risk of having pregnancy-related LPP after childbirth. This study aimed to investigate the association between body perception, pain intensity, and disability in women with pregnancy-related LPP during late pregnancy and postpartum, and to study whether a disturbed body perception during late pregnancy predicted having postpartum LPP.

**Methods:**

A prospective cohort study in 130 primiparous women (median age = 30 years) was performed. Pain intensity, disability, and lumbopelvic body perception during the last month of pregnancy and 6 weeks postpartum were assessed with the Numerical Pain Rating Scale (NPRS), Oswestry Disability Index, and Fremantle Back Awareness Questionnaire, respectively. Having pregnancy-related LPP was defined as an NPRS score ≥ 1/10. At both timepoints, women were categorized into three groups; pain-free, LPP with low disability, and LPP with high disability (based on Oswestry Disability Index scores). At each timepoint, body perception was compared between groups, and correlations between body perception, pain intensity, and disability were evaluated in women with LPP by using non-parametric tests. Logistic regression analysis was used to determine whether body perception during the last month of pregnancy predicted the presence of LPP 6 weeks postpartum.

**Results:**

Women with LPP at the end of pregnancy, and 6 weeks postpartum reported a more disturbed body perception compared to pain-free women (*p* ≤ 0.005). Greater body perception disturbance correlated with higher pain intensity (σ = 0.266, *p* = 0.008) and disability (σ = 0.472, *p* < 0.001) during late pregnancy, and with pain intensity 6 weeks postpartum (σ = 0.403, *p* = 0.015). A disturbed body perception during late pregnancy nearly significantly predicted having postpartum LPP (Odds Ratio = 1.231, *p* = 0.052).

**Conclusions:**

Body perception disturbance was greater in women experiencing LPP during late pregnancy and postpartum compared to pain-free women, and correlated with pain intensity and disability. Though non-significant (*p* = 0.052), the results of the regression analysis suggest that greater body perception disturbance during late pregnancy might predict having LPP postpartum. However, future studies should follow up on this.

**Supplementary Information:**

The online version contains supplementary material available at 10.1186/s12884-021-03704-w.

## Background

Lumbopelvic pain (LPP), or musculoskeletal pain at the lower back and/or pelvic girdle, affects 50–90% of pregnant women [1–3]. Hence, pregnancy-related LPP is often considered a normal and inevitable part of pregnancy. However, its negative impact in terms of disability, sick leave, and loss of quality of life should not be underestimated [4, 5]. Though many women with pregnancy-related LPP recover after childbirth [1, 6], 21% continues to report pain 2 to 3 years postpartum [7, 8]. Moreover, 10% still experiences disability, a poorer quality of life, and a decreased ability to work full-time a decade after childbirth [9]. Finally, having LPP during pregnancy has been shown to increase the risk of developing low back and/or pelvic girdle pain during later pregnancies [10], and later life phases [11, 12]. Thus, to reduce the (long-lasting) negative consequences of pregnancy-related LPP, it is crucial to identify women at risk of developing postpartum LPP.

Most often, pregnancy-related LPP does not result from a specific disease or pathoanatomical abnormality. Instead, multiple biological, psychosocial, and lifestyle factors influence the pain and disability experienced [2], similar to other musculoskeletal conditions such as low back pain [13]. Recent studies identified a disturbed body perception as a potential contributor to the pain experience in musculoskeletal conditions [14–16]. Body perception refers to the way we consciously perceive our own body and is considered a dynamic construct that depends on ongoing visual, tactile, and proprioceptive input, and is influenced by psychosocial factors, memory, and beliefs [15].

Body perception disturbances have been observed in patients with chronic low back pain [14]. Studies reported that they perceive their back as expanded, shrunken, vulnerable, or fragile [17–20], and that they exhibit impairments in the mechanisms that support body perception, such as poorer tactile and proprioceptive acuity [21–23], problems with localizing and recognizing tactile stimuli [24, 25], and impaired trunk motor imagery [26, 27]. Patients with low back pain also reported a more altered perceptual awareness of the lower back compared to pain-free controls, which correlated with clinical features, such as pain intensity, pain duration, and disability [14, 28–32].

Recent studies demonstrated that women with pregnancy-related LPP also show a disturbed body perception at the lumbopelvic region [33, 34]. However, findings regarding the association with clinical features were inconsistent. In pregnant women with LPP, the extent of body perception disturbance correlated with pain intensity, but not with disability [34]. In contrast, a more disturbed body perception was only found in women with postpartum LPP who experienced moderate (vs. low) disability, suggesting an association between body perception disturbance and disability [33]. The correlation between body perception and pain intensity was not studied [33].

These exploratory studies underscore the potential impact body perception disturbances can have in women with pregnancy-related LPP [33, 34]. However, the sample sizes were fairly small (*n* < 35) and the cross-sectional design did not allow clarifying the directional pathways between body perception disturbance and pregnancy-related LPP. Understanding which factors predispose women to developing (persistent) LPP is, however, vital to optimize preventative and treatment strategies.

Therefore, our first aim was to examine the association between body perception and clinical features in primiparous women with pregnancy-related LPP during the last month of pregnancy, and 6 weeks postpartum. At both timepoints, (A) we investigated whether women experiencing high disability due to LPP showed a more disturbed body perception compared to women experiencing low disability and pain-free controls, and (B) we examined the correlation between body perception and clinical pain features in women with LPP. Similar to the general population with low back pain, we hypothesized that a more disturbed body perception in women with pregnancy-related LPP during the end of pregnancy, and 6 weeks postpartum correlates with a higher pain intensity and disability. The second aim of this study was to examine whether a disturbed body perception at the end of pregnancy predicted the presence of postpartum LPP.

## Methods

A cohort study using a convenience sample was performed. Dutch-speaking women who had just delivered their first child (“primiparous women”) at the Maternity Unit of the University Hospitals Leuven, Belgium between August 2016 and December 2016 were asked to participate. In Belgium, more than 98% of women give birth in a hospital rather than at home. Exclusion criteria were: (a history of) neurological disorders or pelvic floor surgery; impaired cognition; multiple gestation pregnancy; and having given birth to a stillborn child, or a child with a mental or physical disorder. Each year, approximately 1265 primiparous women deliver their child at the University Hospitals Leuven, yielding a pool of 527 women potentially eligible for participation during our five-month recruitment period, not considering exclusion criteria. Participants were recruited during this relatively short time period to reduce variation in hospital organization (shortly after recruitment stopped, the hospital stay for a normal delivery in Belgium was shortened by 1 day). The study protocol conformed to the principles of the Declaration of Helsinki (1964) and was approved by the local Ethics Committee Research of UZ/KU Leuven, Belgium (S59108). All subjects provided written informed consent prior to participation. The STROBE guidelines were used to ensure the reporting of this cohort study [35].

We included 130 women. Maximally 3 days after childbirth, following information was collected retrospectively: (1) average intensity of pregnancy-related LPP during pregnancy with the Numerical Pain Rating Scale (NPRS), ranging from 0 = ‘no pain’ to 10 = ‘worst pain’ [36, 37], (2) disability due to LPP during the last 4 weeks of pregnancy with the validated Dutch version of the Oswestry Disability Index (version 2.1.a) (ODI-2) [38] (for English version, see Fairbank et al. [39]), and (3) body perception at the lumbopelvic region during the last four weeks of pregnancy with the validated Dutch version of the Fremantle Back Awareness Questionnaire (FreBAQ) [31] (for English version, see [28]). The ODI-2 evaluates the level of disability due to low back pain during daily life activities, with higher scores indicating higher disability levels [39]. The FreBAQ questions how often (0 = ‘Never’ to 4 = ‘Always’) one experiences symptoms resembling neglect (items 1–3), a reduced lumbar proprioceptive acuity (items 4–5), and alterations in the perceived shape and size of the trunk (items 6–9), with higher scores indicating a more disturbed body perception. Finally, pre-pregnancy body weight, gestational weight gain, and whether women had an operative Caesarean section delivery were retrieved from medical records.

Six weeks postpartum, women were invited to complete the NPRS, ODI-2, and FreBAQ surveys again via a secure online platform for patients (www.nexuzhealth.be). Non-respondents were reminded by email and subsequently by a telephone call within 1 week.

### Statistical analysis

Statistical analyses were performed with SPSS (version 25, IBM, USA). The significance level was set at α = 0.05. Women with an NPRS score exceeding 0/10 during pregnancy, and 6 weeks postpartum were categorized as having ‘prenatal LPP’ and ‘postpartum LPP’, respectively. Women with an NPRS score of 0/10 were considered pain-free.

#### Last month of pregnancy

For the retrospective analysis regarding the last month of pregnancy, women were categorized into three groups (cfr. methodology of Beales et al. [33]). The first group comprised pain-free women (*n* = 31). Women with prenatal LPP were divided into two disability groups by performing a median-split analysis based on the ODI-2 scores of all women with prenatal LPP (median ODI-2 = 16/100). Forty-nine women were assigned to a ‘minimal disability’ group, 50 women to a ‘moderate disability’ group. These labels were based on the actual median ODI-2 scores observed in each group after performing the median-split analysis (resp. 6/100, 29/100) and the ODI-2 categories proposed by Fairbank et al. [39].

Given the non-normal distribution of the variables age and pre-pregnancy body weight (verified with Shapiro-Wilk test: age: No LPP: *p* = 0.074, Minimal disability: *p* = 0.024, Moderate disability: *p* = 0.456; pre-pregnancy body weight: No LPP: *p* = 0.107, Minimal disability: *p* = 0.211, Moderate disability: *p* < 0.001) and ordinal nature of NPRS, ODI-2, and FreBAQ scores, Kruskal-Wallis tests compared age, pre-pregnancy body weight, body perception, and disability between the three groups. In case of a significant result, post-hoc Mann-Whitney U tests with Bonferroni correction (adjusted α = 0.05/3) for pairwise comparisons were performed. Given the normal distribution of the variable gestational weight gain (Shapiro-Wilk test: No LPP: *p* = 0.663, Minimal disability: *p* = 0.769, Moderate disability: *p* = 0.093), a one-way analysis of variance compared gestational weight gain between the three groups, followed by post-hoc independent t-tests with Bonferroni correction (adjusted α = 0.05/3) for pairwise comparisons. NPRS scores between the two subgroups with prenatal pregnancy-related LPP were compared with a Mann-Whitney U test. Finally, correlations between body perception, pain intensity, and disability were evaluated in the pooled group of women with prenatal LPP by using Spearman’s correlation tests. 95% confidence intervals (95% CI) around the Spearman’s σ-values were based on the Fisher r-to-z transformation.

#### Six weeks postpartum

At 6 weeks postpartum, participants were divided into three groups by using the above-described method. The first group consisted of the pain-free women (*n* = 18). A median-split analysis based on ODI-2 scores (median ODI-2 = 3/100) divided the women with postpartum LPP into a group with ‘no disability’ (*n* = 18) and a group with ‘minimal disability’ (*n* = 18). These labels were based on the actual median ODI-2 score in each group after the median-split analysis was performed (respectively 0/100 and 10/100).

Given the non-normal distribution of age in the three groups postpartum (Shapiro-Wilk test: No LPP: *p* = 0.036, No disability: *p* = 0.024, Minimal disability: *p* = 0.615), and the ordinal nature of FreBAQ and ODI-2 scores, age, body perception, and disability at 6 weeks postpartum were compared between the three groups with Kruskal-Wallis tests, followed by post-hoc Mann-Whitney U tests with Bonferroni correction (adjusted α = 0.05/3). A Mann-Whitney U test compared NPRS scores between the two subgroups with postpartum LPP. A Fisher-Freeman-Halton Exact test verified whether the proportion of having had a Caesarean section delivery differed between groups. Finally, Spearman’s correlation tests determined correlations between body perception, pain intensity, and disability in the pooled group of women with postpartum LPP.

#### Logistic regression

Due to a drop-out rate of 58% (see below), the number of cases at 6 weeks postpartum did not allow for a multivariate logistic regression analysis (*n* = 36 women with postpartum LPP, *n* = 18 pain-free women) [40, 41]. A univariate logistic regression analysis determined the Odds Ratio and association between the predictor variable ‘body perception during the last month of pregnancy’ and the presence of LPP 6 weeks postpartum.

## Results

### Last month of pregnancy

Ninety-nine out of 130 women (76.2%) reported pregnancy-related LPP during pregnancy, with 49 women experiencing minimal disability and 50 women moderate disability. No group differences in age, pre-pregnancy body weight, and gestational weight gain were found. However, body perception differed significantly between the three groups (Kruskal-Wallis Test: *p* < 0.001, Table 1). Post-hoc tests revealed that women with LPP and moderate disability exhibited a significantly more disturbed body perception compared to pain-free women and women with minimal disability (Table 1). No differences in body perception were found between women with minimal disability and pain-free women (*p* = 0.089). Women with moderate disability also reported a significantly higher pain intensity compared to the minimal disability group (Table 1).

**Table 1:** Differences between groups during the last month of pregnancy reported as median (interquartile range) or average (± standard deviation)

					Test statistic for pairwise comparison (*p*-value) (ES)		
	No LPP (*n* = 31)	Minimal disability (*n* = 49)	Moderate disability (*n* = 50)	Test statistic (*p*-value)	No LPP - Minimal disability	No LPP - Moderate disability	Minimal disability - Moderate disability
**Age (years)**	31 (29–34)	31 (29–33)	30 (29–32)	1.78^a^ (0.42)	741.5^b^ (0.86)	666.0^b^ (0.29)	1058.0^b^ (0.24)
**Pre-pregnancy weight (kg)**	63 (57–69)	65 (58–73)	62 (57–69)	0.63^a^ (0.73)	678.5^b^ (0.51)	759.0^b^ (0.88)	1103.5^b^ (0.49)
**Weight gain (kg)**	14.0 (± 4.5)	14.6 (± 4.3)	16.1 (± 6.2)	1.761^c^ (0.18)	−5.97^d^ (0.55)	−1.59^d^ (0.12)	−1.35^d^ (0.18)
**NPRS LPP**	0 (0–0)	3 (2–4)	6 (4–7)	NA	NA	NA	**592.5 (< 0.001) (−0.45)**
**ODI-2**	2 (0–10)	6 (2–12)	29 (22–35)	**82.34**^**a**^ **(< 0.001)**	597.5^b^ (0.10)	**114.5**^**b**^ **(< 0.001) (−0.72)**	**0.0**^**b**^ **(< 0.001) (−0.86)**
**FreBAQ**	0 (0–1)	0 (0–3)	5 (2–9)	**37.86**^**a**^ **(< 0.001)**	608.5^b^ (0.09)	**248.0**^**b**^ **(< 0.001) (−0.58)**	**547.5**^**b**^ **(< 0.001) (−0.49)**

Significant *p*-values are highlighted in bold. Effect size for between-group differences are reported for statistically significant results

*Abbreviations*: *LPP* lumbopelvic pain, *NPRS* Numerical Pain Rating Scale (0–10), *ODI-2* Oswestry Disability Index - version 2 (0–100), *FreBAQ* Fremantle Back Awareness Questionnaire (0–36), *ES* effect size, *NA* not applicable

^a^Kruskal-Wallis H-value

^b^Mann-Whitney U-value

^c^F-value

^d^t-value

To better understand the different constructs of body perception, Additional file 1 presents the frequency of responding to each FreBAQ item in every group. Pain-free women reported some disturbances in body perception. The items they endorsed to some extent (i.e., score ≥ 1) most frequently were item 5 (position of back unknown), item 9 (back feels lopsided), and item 6 (unable to perceive outline of back), endorsed by respectively 19.3, 16.1, and 13.0% of pain-free women. Women with minimal disability most frequently endorsed item 5 (back position unknown, 24.5%), item 4 (back movement unknown, 21.3%), and item 6 (unable to perceive outline, 20.8%) to some extent, while women with moderate disability most often endorsed item 5 (back position unknown, 64.0%), item 9 (back feels lopsided, 54.2%), and item 4 (back movement unknown, 46.0%) to some extent. Note that the frequencies of assigning a score of ≥ 1 to these items was markedly higher in the moderate disability group compared to the other groups (e.g., item 5: 64.0% compared to 19.3% and 24.5%).

**Fig. 1:**
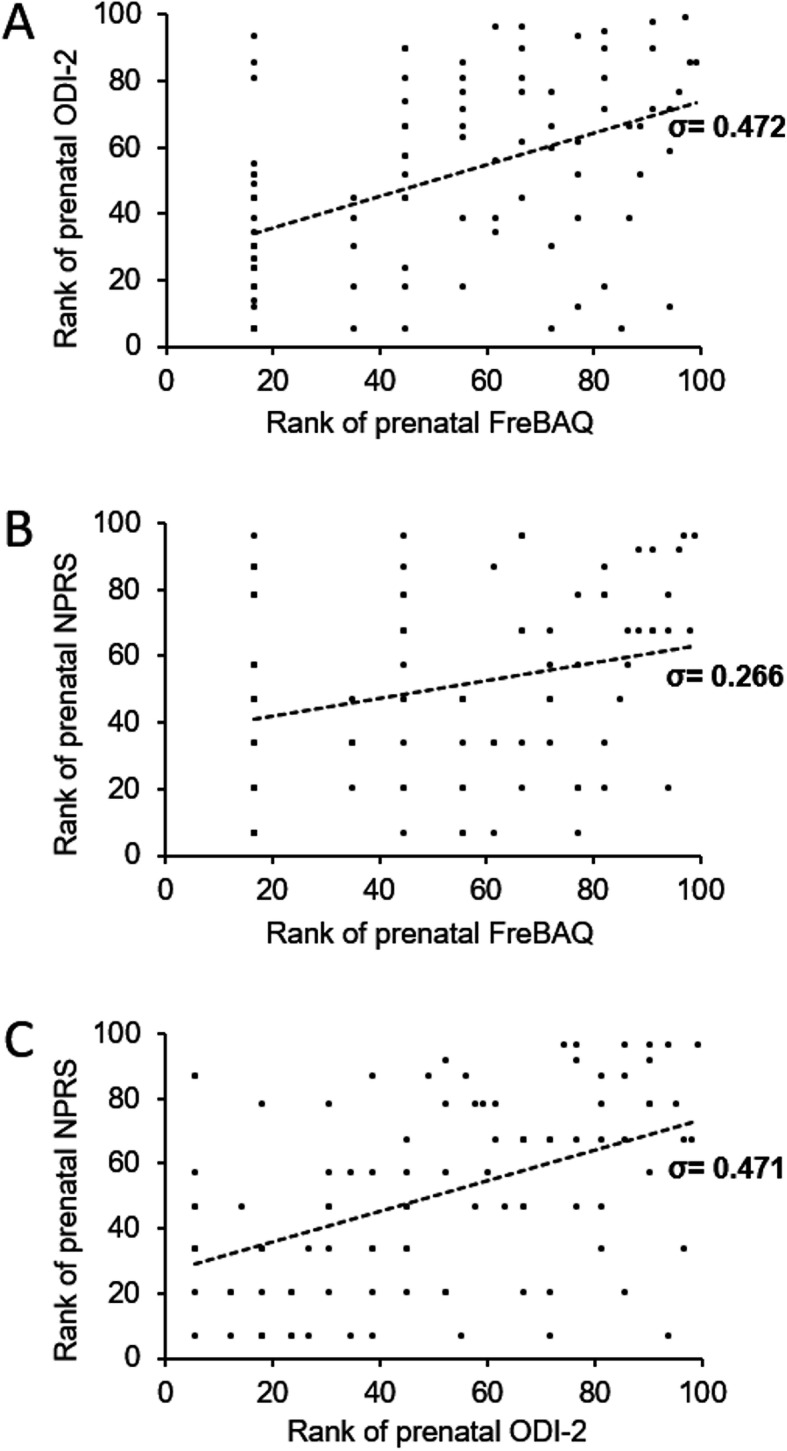
Scatter plots of ranked **a** FreBAQ and ODI-2 scores, **b** FreBAQ and NPRS scores, **c** ODI-2 and NPRS scores, and Spearman’s σ, in women with pregnancy-related LPP at the end of pregnancy

When women with prenatal LPP were pooled, a more disturbed body perception correlated significantly with a higher disability (σ = 0.472, 95% CI = 0.293 to 0.619, *p* < 0.001) and a higher pain intensity (σ = 0.266, 95% CI = 0.069 to 0.443, *p* = 0.008) (Fig. 1). Disability and pain intensity were significantly correlated as well (σ = 0.471, 95% CI = 0.292 to 0.618, *p* < 0.001, Fig. 1).

### Six weeks postpartum

Fifty-four out of 130 women (41.5%) responded at 6 weeks postpartum. Reasons for not-responding were not questioned, but we did verify whether outcome measures assessed at the end of pregnancy differed between women lost to follow-up and women who were not. Because the variables age, pre-pregnancy body weight and gestational weight gain were not-normally distributed (Shapiro-Wilk test: age: Lost to follow-up: *p* = 0.018, Not lost to follow-up: *p* = 0.012; pre-pregnancy body weight: Lost to follow-up: *p* < 0.001, Not lost to follow-up: *p* = 0.003; gestational weight gain: Lost to follow-up: *p* = 0.018, Not lost to follow-up: *p* = 0.559), and due to the ordinal nature of the NPRS, ODI-2 and FreBAQ scores, Mann-Whitney U tests determined whether these outcomes differed between groups. No between-group differences in pre-pregnancy body weight and body perception during the last month of pregnancy were found. However, women lost to follow-up had gained significantly more weight and reported a significantly higher pain intensity, as well as a trend towards significantly more disability during the last month of pregnancy compared to women who responded 6 weeks postpartum (Table 2).

**Table 2:** Differences in outcomes during late pregnancy between women lost to follow-up and women who were not

	Lost to follow-up (*n* = 76)	Not lost to follow-up (*n* = 54)	Mann-Whitney U (*p*-value) (ES)
**Age (years)**	30 (28–32)	31 (29–33)	*1678.5 (0.07) (−0.16)*
**Pre-pregnancy weight (kg)**	63 (58–70)	61.5 (56–69)	1770.0 (0.22)
**Gestational weight gain (kg)**	16 (13–19)	14 (11–17)	**1527.5 (0.03) (−0.19)**
**NPRS LPP (0–10)**	3 (1–6)	2 (0–5)	**1621.0 (0.04) (−0.18)**
**ODI-2 (0–100)**	16 (4–29)	8 (3–20)	*1643.0 (0.05) (−0.17)*
**FreBAQ (0–36)**	2 (0–6)	2 (0–5)	1907.0 (0.46)

Values reported as median (interquartile range)

Significant *p*-values (*p* < 0.05) are highlighted in bold, nearly significant *p*-values (0.05 < *p* < 0.10) in italics. Effect size for between-group differences are reported for statistically and nearly statistically significant results

*Abbreviations*: *NPRS* Numerical Pain Rating Scale, *LPP* lumbopelvic pain, *ODI-2* Oswestry Disability Index - version 2, *FreBAQ* Fremantle Back Awareness Questionnaire, *ES* effect size

Thirty-six out of 54 women (66.7%) reported pregnancy-related LPP at 6 weeks postpartum. Eighteen women experienced no disability and 18 women reported minimal disability. The three groups did not differ in terms of age and the proportion of having had a Caesarean section delivered (Table 3), but significant between-group differences in body perception were found (Kruskal-Wallis test, *p* < 0.001). Post-hoc tests revealed that women experiencing minimal disability and women experiencing no disability due to LPP reported a significantly more disturbed body perception compared to pain-free women at 6 weeks postpartum (Table 3). However, body perception did not differ between the two groups with LPP (*p* = 0.208). Women with minimal disability also reported a significantly higher pain intensity compared to women with no disability (*p* = 0.002).

**Table 3:** Differences between groups at 6 weeks postpartum, reported as median (interquartile range)

					Test statistic for pairwise comparison (*p*-value) (ES)		
	No LPP (*n* = 18)	Non-disabling LPP (*n* = 18)	Minimally disabling LPP (*n* = 18)	Test statistic (*p*-value)	No LPP - Non-disabling LPP	No LPP - Minimally disabling LPP	Non-disabling LPP - Minimally disabling LPP
**Age (years)**	31 (29–33)	31 (29–33)	32 (30–34)	1.54^a^ (0.46)	159.0^b^ (0.92)	128.5^b^ (0.29)	128.5^b^ (0.28)
**C-section (yes/no)**	3/15	3/15	7/11	2.96^c^ (0.24)	NA	NA	NA
**NPRS LPP**	0 (0–0)	1 (1–2)	4 (4–6)	NA	NA	NA	**66.5**^**b**^ **(0.002) (−0.52)**
**ODI-2**	0 (0–0)	0 (0–1)	10 (8–16)	**41.95**^**a**^ **(< 0.001)**	128.5^b^ (0.10)	**5.0**^**b**^ **(< 0.001) (−0.88)**	**0.0**^**b**^ **(< 0.001) (−0.88)**
**FreBAQ**	0 (0–0)	2 (0–5)	3.5 (0–10)	**13.24**^**a**^ **(0.001)**	**85.0**^**b**^ **(0.005)**	**63.0**^**b**^ **(0.001) (0.57)**	123.0^b^ (0.21)

*Abbreviations*: *LPP* lumbopelvic pain, *C-section* Caesarean section delivery, *NPRS* Numerical Pain Rating Scale (0–10), *ODI-2* Oswestry Disability Index – version 2 (0–100), *FreBAQ* Fremantle Back Awareness Questionnaire (0–36), *ES* effect size, *NA* not applicable

Significant *p*-values highlighted in bold. Effect size for between-group differences are reported for statistically significant results

^a^Kruskal-Wallis H-value

^b^Mann-Whitney U-value

^c^Fisher-Freeman-Halton Exact test value

Additional file 2 presents the frequency of responding to each FreBAQ item 6 weeks postpartum. Pain-free women reported some body perception disturbances, most frequently endorsing item 5 (back position unknown, 16.7%), item 4 (back movement unknown, 11.1%), and item 6 (unable to perceive outline, 5.6%). Women with non-disabling postpartum LPP most frequently endorsed item 5 (back position unknown, 47.2%), item 6 (unable to perceive outline, 33.3%), and item 9 (back lopsided, 36.1%), while those with minimal disability most often endorsed the same items but at a higher frequency, i.e., item 5 (55.6%), item 6 (44.4%), and item 9 (50%).

**Fig. 2:**
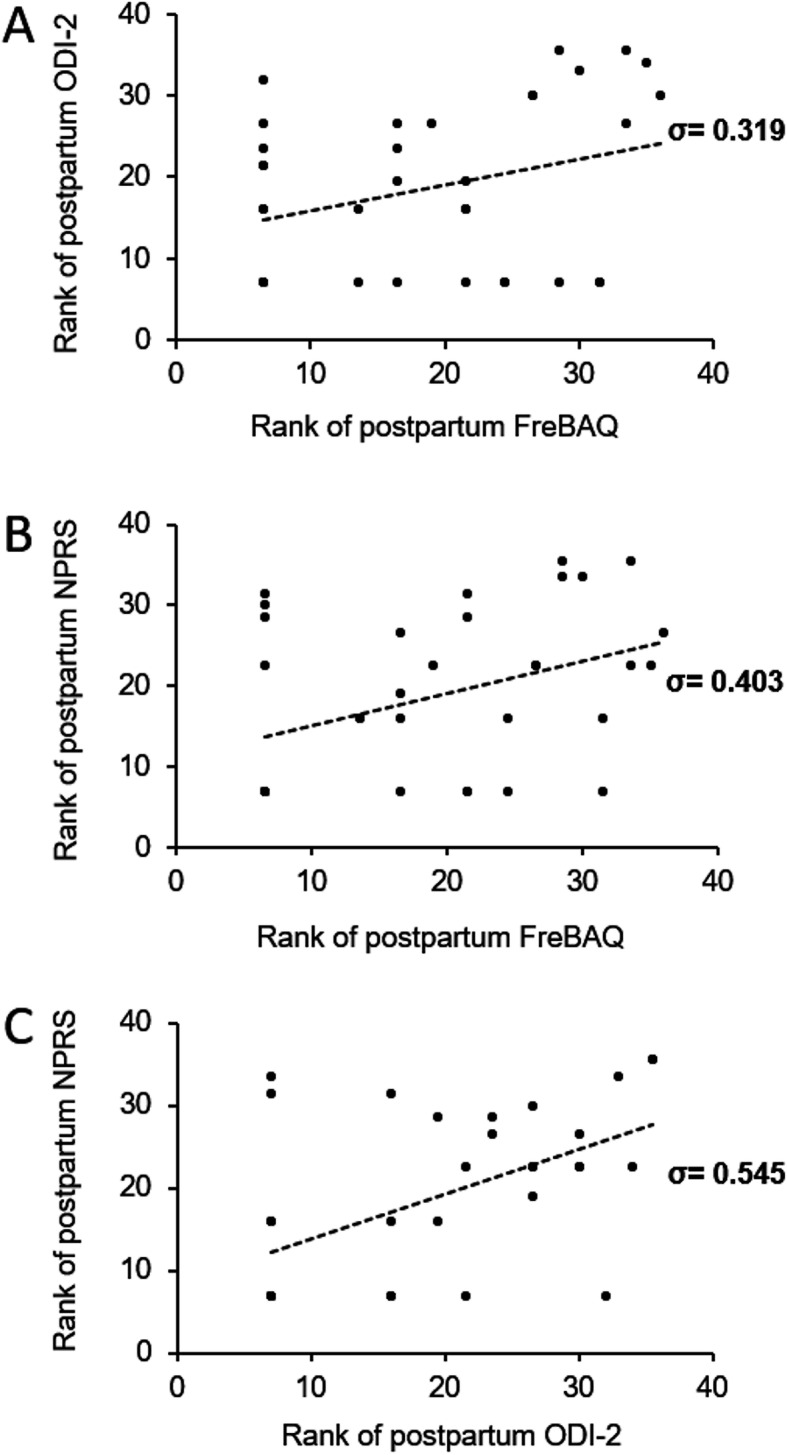
Scatter plots of ranked **a** FreBAQ and ODI-2 scores, **b** FreBAQ and NPRS scores, **c** ODI-2 and NPRS scores, and Spearman’s σ in women with pregnancy-related LPP 6 weeks postpartum

In the pooled group of women with postpartum LPP, a more disturbed body perception correlated significantly with a higher pain intensity (σ = 0.403, 95% CI = 0.072 to 0.654, *p* = 0.015) and showed a trend towards a correlation with disability (σ = 0.319, 95% CI = − 0.019 to 0.592, *p* = 0.058) (Fig. 2). Disability and pain intensity were correlated as well (σ = 0.545, 95% CI = 0.241 to 0.752, *p* < 0.001) (Fig. 2).

### Prediction of postpartum LPP based on FreBAQ scores at the end of pregnancy

A higher FreBAQ score at the end of pregnancy showed a trend towards being significantly predictive for having LPP at 6 weeks postpartum (*p* = 0.052) (Table 4). This nearly significant result suggests that for every point the FreBAQ score increased, the risk of having postpartum LPP tended to increase by 23.1%.

**Table 4:** Univariate logistic regression model predicting whether a participant experienced LPP at 6 weeks postpartum

	B (SE)	Wald	Odds ratio	95% CI for odds ratio	*p*-value
**FreBAQ **	0.207 (0.107)	3.762	1.231	0.998–1.518	*0.052*

*N* = 54 women 6 weeks postpartum. *R*^*2*^ = 0.095 (Cox & Snell), *R*^*2*^ = 0.132 (Naegelkerke), χ^2^(4) = 2.388, *p* > 0.05 (Hosmer & Lemeshow)

Nearly significant *p*-value (0.05 < *p* < 0.10) highlighted in italics

*Abbreviations*: *LPP* lumbopelvic pain, *FreBAQ* Fremantle Back Awareness Questionnaire (0-36), *B* beta value, *SE* standard error, *CI* confidence interval

## Discussion

This is the first longitudinal study that investigated (1) differences in body perception at the lumbopelvic region between women experiencing low and high disability due to pregnancy-related LPP, and the association with clinical features during the last month of pregnancy and 6 weeks postpartum, and (2) whether body perception disturbances at the end of pregnancy predict the presence of postpartum LPP. We found that women with moderate disability due to pregnancy-related LPP during late pregnancy reported a more disturbed body perception compared to women with minimally disabling LPP and pain-free women. At 6 weeks postpartum, a disturbed body perception was found in all women with pregnancy-related LPP, regardless of their disability level. Finally, we found that an altered body perception during late pregnancy was nearly significantly predictive of having LPP at 6 weeks postpartum (*p* = 0.052). Though these latter results need confirmation in a larger sample of postpartum women, allowing for multivariate regression analyses, our findings suggest that body perception disturbances might play a role in the persistence of pregnancy-related LPP postpartum. Hence, it might be important to address body perception disturbances in pregnant women, regardless of whether they (already) have pregnancy-related LPP. Future studies should also investigate whether interventions that address body perception disturbances and have shown potential in patients with chronic low back pain free from pregnancy [42–45], are valuable in women with pregnancy-related LPP and/or body perception disturbances.

In this study, 76.2% of pregnant women reported LPP, which aligned with previously observed prevalence rates ranging from 68 to 86% [1, 46–48]. To the best of our knowledge, only one study investigated pain in the lumbopelvic region (i.e., lower back, pelvic girdle or upper back pain, and overlapping pain constellations) in the early postpartum period (i.e., first 6 to 10 weeks after childbirth) [49]. Up to 67% reported low back pain and/or pelvic girdle pain, with or without upper back pain [49], which accords with the prevalence rate we observed (66.7%). To note, the median ODI-2 score of women with postpartum LPP in our study was 3/100. Thus, though the majority of women experienced postpartum LPP, its impact on daily functioning was limited. Nevertheless, four out of 36 women (11.1%) reported moderate to severe disability (ODI-2 = 20 to 54/100). These higher disability levels might be particularly challenging to address, especially when they become persistent, and should therefore not be underestimated.

Compared to pain-free women, women with LPP during pregnancy and postpartum reported a more disturbed body perception, which correlated with higher pain intensities. These results corroborate previous findings of an altered body perception in women with pregnancy-related LPP and patients with chronic low back pain [28–34], and highlight the impact of body perception disturbances on the pain experience. A disturbed body perception might, for instance, induce pain through a mismatch between predicted and actual responses of motor actions [50]. Second, as the intact perception of one’s body and the space surrounding it is crucial for optimal movement, a disturbed body perception might compromise trunk movement quality, leading to abnormal tissue loading and related pain [51]. This is supported by the fact that the most frequently endorsed FreBAQ items by women with pregnancy-related LPP were those related to a reduced proprioceptive acuity and to the feeling of having an asymmetrical back. Finally, changes in body perception have been shown to be related to pain- and movement-related fear and pain catastrophizing in subjects with pregnancy-related LPP and chronic low back pain [28, 29, 32–34]. According to the Fear Avoidance Model, these maladaptive cognitions and beliefs negatively affect pain intensity, disability, and the persistence of LPP postpartum [52, 53].

The association between body perception and disability was less straight-forward and seemed to differ between timepoints. During late pregnancy, significantly increased FreBAQ scores were only found in women reporting moderate (rather than minimal) disability, and FreBAQ scores correlated significantly with disability. This aligned with the findings of Wand et al. [34]. However, at 6 weeks postpartum, body perception was disturbed in all women with pregnancy-related LPP, regardless of their disability levels. This was in contrast to the study of Beales et al. [33], demonstrating that only women with moderate (vs. mild) disability reported higher FreBAQ scores compared to pain-free subjects. Moreover, in our study, FreBAQ scores and ODI-2 scores only tended to correlate at 6 weeks postpartum, which might be due to the fact that disability levels observed at that timepoint were markedly lower (13 out of 36 women had an ODI-2 score of 0/100) compared to those reported in Beales et al. [33]. A potential reason for this is that women with higher pain intensity and disability at the end of pregnancy seemed to drop out of this study. Future studies involving a larger sample of postpartum women with LPP and higher disability levels might further elucidate the association between an altered body perception and disability in women with postpartum LPP.

The median FreBAQ scores reported by pain-free women at both timepoints were similar to those observed in previous studies in healthy men and women (0/36) [28], and pain-free pregnant (1/36) and postpartum women (2/36) [33, 34]. This seems to suggest that pain-free women do not exhibit an altered body perception, despite the sudden large changes in body shape and size during pregnancy and after childbirth. Nevertheless, some pain-free women did report body perception disturbances. At the end of pregnancy, 20% reported a reduced lumbar movement sense and 16% perceived their back as asymmetrical. At 6 weeks postpartum, 17% of pain-free women experienced lumbar proprioceptive impairments (i.e., decreased position and movement sense). These findings suggest that some women do develop body perception disturbances during pregnancy, possibly via changes in body schema and/or body weight during pregnancy [54–57]. However, as we did not evaluate body perception during early pregnancy or before conception, we cannot exclude that body perception disturbances were already present before gestation. Further longitudinal studies examining body perception from pre-conception to postpartum might elucidate whether this feature alters during pregnancy.

We also found that a more disturbed body perception at the end of pregnancy tended to predict the presence of LPP 6 weeks postpartum. Previous studies already identified a history of low back pain, pre-pregnancy body mass index > 25 kg/m^2^, pelvic girdle pain, depression, and a heavy workload in pregnancy as predictive factors for postpartum LPP [58]. However, these studies did not investigate body perception. Our regression analysis was borderline significant (*p* = 0.052), possibly due to the high drop-out rate, lowering statistical power. Therefore, caution is warranted when interpreting this result. Moreover, because we did not evaluate women before or at the beginning of pregnancy, it remains unclear whether body perception disturbances are either a cause or consequence of pregnancy-related LPP. Finally, because we could not perform multivariate regression analyses, it remains unclear whether the predictive value of a disturbed body perception at the end of pregnancy could be partly explained through its correlation with pain intensity and disability. Still, our findings underpin the importance of assessing body perception in pregnant women, even if they do not report LPP, and of addressing body perception disturbances with targeted treatment interventions, such as sensorimotor retraining, graded motor imagery, or seeing the back during movement [42, 44, 59], to prevent the persistence of LPP postpartum.

Finally, we would like to emphasize the importance of (pro)actively assessing body perception during pregnancy, even when women do not mention such disturbances themselves. Individuals experiencing body perception disturbances have been shown to be apprehensive of discussing this with healthcare professionals and significant others [60], as they fear not being believed or because the “bizarre” nature of their experience suggests that they might have a mental disorder [61, 62]. Consequently, experiencing body perception disturbances might increase stress, pain, and fear-avoidance behaviour, driving the vicious circle towards more pain and disability.

### Limitations

The results of this study need to be considered in light of its limitations. Though this is the first longitudinal study evaluating body perception during pregnancy until postpartum, we did not assess women before or at the beginning of pregnancy. Therefore, it remains unclear whether women with body perception disturbances, irrespective of having pregnancy-related LPP, are predisposed to developing (persistent) LPP. Second, future studies using physical measures of body schema or body representation, such as repositioning accuracy [63], sensory localization [25], or sensory dissociation [64], would further improve our understanding of body perception disturbances in pregnancy-related LPP. Third, 58.5% of the participants, and in particular those with higher pain intensity and disability at the end of pregnancy, dropped out after childbirth. Since greater pain intensity and disability during pregnancy have been shown to predict postpartum LPP [65, 66], our regression analysis might have returned different results to the ones we observed. Reasons for loss to follow-up were not questioned, but they might relate to the fact that we included primiparous women. Becoming a first-time-mother is lifechanging. Hence, their commitment to continuing participation might have been lower compared to multiparous women. Fourth, women with pregnancy-related LPP were divided into disability groups by using a median-split analysis rather than the categories proposed by Fairbank et al. [39]. However, we chose to use the same approach as Beales et al. [33]. Fifth, there is currently no consensus regarding the diagnosis of pregnancy-related LPP and this could have influenced the observed number of cases with LPP. We chose to define pregnancy-related LPP as self-reported pain (i.e., NPRS≥1/10) in the lumbopelvic region comprising the lumbar spine and anterior and posterior region of the pelvic girdle, in line with Olsson et al. [67]. Sixth, maximally 3 days after childbirth, participants retrospectively evaluated pain intensity, disability and body perception experienced during the last month of pregnancy. For pain intensity and disability, recall periods of up to 3 months have shown acceptable validity [68, 69]. However, we cannot exclude that just having given birth might have affected these ratings. Moreover, the validity of FreBAQ scores concerning the past month has not been investigated. Seventh, there is evidence that women with a hypermobility spectrum disorder (e.g., Generalized Joint Hypermobility, Ehlers-Danlos Syndrome) have higher odds of experiencing LPP during pregnancy [46, 70, 71], and that individuals with a hypermobility spectrum disorder show poorer proprioception, at least at the lower and upper limbs [72]. Hence, joint hypermobility could have driven the association between body perception disturbance and LPP found in our study. Future studies might consider assessing joint hypermobility to elucidate its role in the development and persistence of pregnancy-related LPP. Finally, our predictive model did not include other predictors for postpartum LPP, such as pain intensity, disability, a history of low back pain, BMI > 25 kg/m^2^, or depression [58, 73]. Future studies should include these predictors to determine how much variance in the persistence of pregnancy-related LPP can be explained by body perception disturbances when considering other predictors.

## Conclusions

We demonstrated that body perception at the lumbopelvic region was significantly more disturbed in women with LPP during late pregnancy and 6 weeks postpartum compared to pain-free women. Greater levels of body perception disturbance correlated significantly with higher pain intensity at both timepoints, and with disability at the end of pregnancy. Finally, a more disturbed body perception during late pregnancy tended to predict the presence of LPP in the early postpartum period. Though longitudinal studies comprising the beginning of pregnancy are needed, our findings suggest that body perception should be assessed and addressed with targeted care during pregnancy, regardless of whether women experience pregnancy-related LPP or not.

## Supplementary Information

**Additional file 1.** Frequency of responding to each FreBAQ item at the end of pregnancy (in %).

**Additional file 2.** Frequency of responses to each FreBAQ item at 6 weeks postpartum (in %).

## Data Availability

The datasets used and analysed during the current study are available from the corresponding author on reasonable request.

## Abbreviations

FreBAQ: Fremantle Back Awareness Questionnaire
NPRS: Numerical Pain Rating Scale
ODI-2: Oswestry Disability Index (version 2.1.a)
LPP: Lumbopelvic pain
95% CI: 95% confidence interval
