# Supplementary material for: Body perception disturbances in women with pregnancy-related lumbopelvic pain and their role in the persistence of pain postpartum
Source: BMC Pregnancy Childbirth. 2021 Mar 18;21:219. doi: 10.1186/s12884-021-03704-w (PMC7977601; doi:10.1186/s12884-021-03704-w)
Supplement: Supplementary file 1 — Additional file 1. Frequency of responding to each FreBAQ item at the end of pregnancy (in %). [file 12884_2021_3704_MOESM1_ESM.docx]

| **Additional file 1.** Frequency of responding to each FreBAQ item at the end of pregnancy (in %). | | | | | |
| --- | --- | --- | --- | --- | --- |
|  | **Never (%)** | **Rarely (%)** | **Occasionally (%)** | **Often (%)** | **Always (%)** |
| **No LPP (n= 31)** | | | | | |
| **Item 1** | 96.8 | 3.2 | - | - | - |
| **Item 2** | 96.8 | 3.2 | - | - | - |
| **Item 3** | 96.8 | 3.2 | - | - | - |
| **Item 4** | 93.4 | 3.2 | 3.2 | - | - |
| **Item 5** | 80.7 | 9.6 | 6.5 | 3.2 | - |
| **Item 6** | 87.0 | 6.5 | 6.5 | - | - |
| **Item 7** | 90.3 | 6.5 | 3.2 | - | - |
| **Item 8** | 96.8 | 3.2 | - | - | - |
| **Item 9** | 83.9 | 3.2 | 9.7 | - | 3.2 |
| **Minimally disabling prenatal LPP (n= 49)** | | | | | |
| **Item 1** | 89.8 | 8.2 | 2.0 | - | - |
| **Item 2** | 89.8 | 8.2 | 2.0 | - | - |
| **Item 3** | 95.9 | 2.0 | 2.0 | - | - |
| **Item 4** | 78.7 | 12.8 | 8.5 | - | - |
| **Item 5** | 75.5 | 10.2 | 8.2 | 6.1 | - |
| **Item 6** | 79.2 | 10.4 | 6.3 | 6.1 | - |
| **Item 7** | 93.9 | 4.1 | 2.0 | - | - |
| **Item 8** | 85.7 | 8.2 | 4.1 | - | 2.0 |
| **Item 9** | 79.6 | 10.2 | 6.1 | 2.0 | 2.0 |
| **Moderately disabling prenatal LPP (n= 50)** | | | | | |
| **Item 1** | 75.5 | 14.3 | 8.2 | 2.0 | - |
| **Item 2** | 56.5 | 26.0 | 16.0 | 2.0 | - |
| **Item 3** | 78.0 | 18.0 | 4.0 | - | - |
| **Item 4** | 54.0 | 16.0 | 14.0 | 16.0 | - |
| **Item 5** | 36.0 | 20.0 | 18.0 | 22.0 | 4.0 |
| **Item 6** | 57.1 | 16.3 | 14.3 | 10.2 | 2.0 |
| **Item 7** | 75.5 | 10.2 | 8.2 | 6.1 | - |
| **Item 8** | 66.7 | 12.5 | 14.6 | 2.1 | 4.2 |
| **Item 9** | 45.8 | 16.6 | 29.2 | 6.3 | 2.1 |
| Abbreviations: LPP= lumbopelvic pain, FreBAQ= Fremantle Back Awareness Questionnaire. Item 1: “My back feels as though it is not part of the rest of my body”; Item 2: “I need to focus all my attention on my back to make it move the way I want it to”; Item 3: “I feel as if my back sometimes moves involuntarily, without my control”; Item 4: “When performing everyday tasks, I don’t know how much my back is moving”; Item 5: “When performing everyday tasks, I am not sure exactly what position my back is in”; Item 6: “I can’t perceive the exact outline of my back”; Item 7: “My back feels like it is enlarged (swollen)”; Item 8: “My back feels like it has shrunk”; Item 9: “My back feels lopsided (asymmetrical)”. For the Dutch translation of the items, see Janssens et al. (2017). | | | | | |
