# Supplementary material for: Body perception disturbances in women with pregnancy-related lumbopelvic pain and their role in the persistence of pain postpartum
Source: BMC Pregnancy Childbirth. 2021 Mar 18;21:219. doi: 10.1186/s12884-021-03704-w (PMC7977601; doi:10.1186/s12884-021-03704-w)
Supplement: Supplementary file 2 — Additional file 2. Frequency of responses to each FreBAQ item at 6 weeks postpartum (in %). [file 12884_2021_3704_MOESM2_ESM.docx]

| **Additional file 2.** Frequency of responses to each FreBAQ item at six weeks postpartum (in %). | | | | | |
| --- | --- | --- | --- | --- | --- |
|  | **Never (%)** | **Rarely (%)** | **Occasionally (%)** | **Often (%)** | **Always (%)** |
| **No postpartum LPP (n= 18)** | | | | | |
| **Item 1** | 100 | - | - | - | - |
| **Item 2** | 100 | - | - | - | - |
| **Item 3** | 94.4 | 5.6 | - | - | - |
| **Item 4** | 88.9 | 5.6 | 5.6 | - | - |
| **Item 5** | 83.3 | 5.6 | 11.1 | - | - |
| **Item 6** | 94.4 | 5.6 | - | - | - |
| **Item 7** | 100 | - | - | - | - |
| **Item 8** | 100 | - | - | - | - |
| **Item 9** | 100 | - | - | - | - |
| **Non-disabling postpartum LPP (n= 18)** | | | | | |
| **Item 1** | 75.0 | 13.9 | - | - | 11.1 |
| **Item 2** | 83.3 | 13.9 | - | 2.8 | - |
| **Item 3** | 88.9 | 11.1 | - | - | - |
| **Item 4** | 77.8 | 8.3 | 5.6 | 8.3 | - |
| **Item 5** | 52.8 | 19.4 | 22.2 | 5.6 | - |
| **Item 6** | 66.7 | 13.9 | 16.7 | - | 2.8 |
| **Item 7** | 83.3 | 5.6 | 11.1 | 2.8 | - |
| **Item 8** | 80.6 | 5.6 | 11.1 | 2.8 | - |
| **Item 9** | 63.9 | 11.1 | 16.7 | 8.3 | - |
| **Minimally disabling postpartum LPP (n= 18)** | | | | | |
| **Item 1** | 66.7 | 27.8 | - | - | 5.6 |
| **Item 2** | 72.2 | 22.2 | - | 5.6 | - |
| **Item 3** | 83.3 | 16.7 | - | - | - |
| **Item 4** | 8.3. | 5.6 | 5.6 | 5.6 | - |
| **Item 5** | 44.4 | 22.2 | 22.2 | 11.1 | - |
| **Item 6** | 55.6 | 22.2 | 16.7 | - | 5.6 |
| **Item 7** | 72.2 | 11.1 | 16.7 | - | - |
| **Item 8** | 66.7 | 11.1 | 16.7 | 5.6 | - |
| **Item 9** | 50.0 | 16.7 | 22.2 | 11.1 | - |
| Abbreviations: LPP= lumbopelvic pain, FreBAQ= Fremantle Back Awareness Questionnaire. Item 1: “My back feels as though it is not part of the rest of my body”; Item 2: “I need to focus all my attention on my back to make it move the way I want it to”; Item 3: “I feel as if my back sometimes moves involuntarily, without my control”; Item 4: “When performing everyday tasks, I don’t know how much my back is moving”; Item 5: “When performing everyday tasks, I am not sure exactly what position my back is in”; Item 6: “I can’t perceive the exact outline of my back”; Item 7: “My back feels like it is enlarged (swollen)”; Item 8: “My back feels like it has shrunk”; Item 9: “My back feels lopsided (asymmetrical)”. For the Dutch translation of the items, see Janssens et al. (2017). | | | | | |
